# Pharmaceutical Particulates and Membranes for the Delivery of Drugs and Bioactive Molecules

**DOI:** 10.3390/pharmaceutics12050412

**Published:** 2020-05-01

**Authors:** Diganta B. Das, Mostafa Mabrouk, Hanan H. Beherei, G. Arthanareeswaran

**Affiliations:** 1 Department of Chemical Engineering, Loughborough University, Loughborough LE113TU, Leicestershire, UK; 2 Refractories, Ceramics and Building Materials Department, National Research Centre, 33 El Bohouth St (Former EL Tahrir St), Dokki, Giza P.O.12622, Egypt; mostafamabrouk.nrc@gmail.com (M.M.); hananh.beherei@gmail.com (H.H.B.); 3 Department of Chemical Engineering, National Institute of Technology, Tiruchirappalli 620015, Tamil Nadu, India; arthanareeg@nitt.edu

The delivery of drugs and bioactive molecules using pharmaceutical particulates and membranes are of great significance for various applications such as the treatment of secondary infections, cancer treatment, skin regeneration, orthopedic applications and others. Several techniques can be utilized for the preparation of the particulates and membranes, which include, but are not limited to, lyophilization, microemulsification, nano-spray-drying, nano-electro-spinning, slip casting and 3D printing. The particulates and membranes possess great prospects to improve the existing strategies and develop new ones for sustained and controlled drug delivery. Therefore, the development of this subject as an area of research has been rapid in the last two decades, and their applications are now very broad. However, these depend on the properties of the designed particulates and membranes.

In addressing these points, this Special Issue (SI) of the journal *Pharmaceutics* seeks to highlight the recent trends and innovative developments in the pharmaceutical particulates and membranes for the delivery of drug and bioactive molecules.

We received in total twenty submissions for the SI, all of which went through a rigorous peer review process. Eight papers were declined at the peer review stage, and, the remaining twelve papers have now been published, all as open access papers as per the policy of the journal. The published papers are also being compiled as an edited e-book, to be published by MDPI. We introduce the published twelve papers briefly in this guest editorial.

To begin with, we collaborated with other experts in the field of pharmaceutical particulates and membranes to review the current state of inorganic nanoparticulates and nanomembranes based on their design, and the key factors for adjusting their morphology and size for their possible medical applications, especially as drug carrier materials (Mabrouk et al. [1]). A very good example of these points can be seen in the paper by Kumar et al. [2] who have developed a prolonged release device for site-specific delivery of a neuroprotective agent (nicotine). The device has been formulated as a novel reinforced crosslinked composite polymeric system with the potential for intrastriatal implantation for Parkinson’s disease interventions. These have been developed in the form of membranes with minimal rates of matrix degradation and retarding nicotine release. This has led to the zero-order release for 50 days following exposure to simulated cerebrospinal fluid (CSF).

Mora-Espíet et al. [3] have investigated the effects of specific targeting of microparticles on their internalization by cells under fluidic conditions. For this purpose, two isogenic breast epithelial cell lines, one overexpressing the human epidermal growth factor receptor 2 (HER2) oncogene (D492HER2) and highly tumorigenic, and the other expressing HER2 at much lower levels and nontumorigenic (D492) were cultured in the presence of polystyrene microparticles of 1 µm in diameter, biofunctionalized with either a specific anti-HER2 antibody or a nonspecific secondary antibody. The authors have come to conclude that the biofunctionalization of microparticles with a specific targeting molecule remarkably increases their internalization by cells in fluidic culture conditions (simulating the blood stream).

Huang et al. [4] have reported a modified coaxial electrospraying technique, which explored how to create ibuprofen-loaded hydroxypropyl methylcellulose nanoparticles for accelerating the drug dissolution rate. During this process, it was shown that a key parameter, i.e., the spreading angle of atomization could provide a linkage among the working process, the property of generated nanoparticles and their functional performance. They confirmed that the nanoparticle diameter (prepared based on a modified technique) has a profound influence on the drug release performance. It is envisaged that the clear process–property–performance relationship should be useful for optimizing the electrospraying process, and, in turn, for achieving the medicated nanoparticles with desired functional performances. Shah et al. [5] designed and optimized a nano-emulsion-based system to improve therapeutic efficacy of moxifloxacin in ophthalmic delivery. Their findings suggest that optimized nanoemulsion can enhance the therapeutic effect of moxifloxacin and, therefore, it can be used as a safe and effective delivery vehicle for ophthalmic therapy. In addition, Wan et al. [6] developed a novel sustained release pellet of loxoprofen sodium (LXP) by coating a dissolution-rate controlling sublayer containing hydroxypropyl methyl cellulose (HPMC) and citric acid, and a second diffusion-rate controlling layer containing aqueous dispersion of ethyl cellulose (ADEC) on the surface of a LXP conventional pellet in order to compare its performance in vivo with an immediate release tablet (Loxinon^®^). Their results identified both the citric acid (CA) and ADEC as the dissolution- and diffusion-rate controlling materials significantly decreasing the drug release rate. The optimal formulation for a pH-independent drug release in media has been suggested as at a pH above 4.5 and at slightly slow release in acid medium. The pharmacokinetic studies have revealed that a more stable and prolonged plasma drug concentration profile of the optimal pellets has been achieved, with a relative bioavaibility of 87.16% compared with the conventional tablets.

Iglesias et al. [7] have reported the synthesis and characterization of magnetic nanoparticles of two distinct origins, one inorganic (MNPs) and the other biomimetic (BMNPs). The authors have declared that the BMNPs are better suited to be loaded with drug molecules positively charged at neutral pH (notably, doxorubicin for instance) and released at the acidic tumor environment. In turn, MNPs may provide their transport capabilities under a magnetic field. However, in this study the authors have used a mixture of both kinds of particles at two different concentrations, trying to derive the best from each of them. Also, they have studied which mixture performs better from different points of view, considering factors such as stability and magnetic hyperthermia response, while keeping suitable drug transport capabilities. The authors have recommended this as a close to ideal drug vehicle with enhanced hyperthermia response. Savin et al. [8] have discussed the antitumoral potential of three gel formulations loaded with carbon dots prepared from N-hydroxyphthalimide (CD-NHF) on two types of skin melanoma cell lines as well as two types of breast cancer cell lines in 2D (cultured cells in normal plastic plates) and 3D (Matrigel) models. Antitumoral gels based on sodium alginate (AS), carboxymethyl cellulose (CMC), and the carbomer Ultrez 10 (CARB) loaded with CD-NHF. The in vitro results for the tested CD-NHF-loaded gel formulations have revealed that the new composites can affect the number, size, and cellular organization of spheroids and impact individual tumor cell ability to proliferate and aggregate in spheroids.

Guadarrama-Acevedo et al. [9] have prepared a novel biodegradable wound dressing by means of alginate membrane and polycaprolactone nanoparticles loaded with curcumin for potential use in wound healing. The membrane has exhibited a diverse range of functional characteristics required to perform as a substitute for synthetic skin, such as a high capacity for swelling and adherence to the skin, evidence of pores to regulate the loss of transepidermal water, transparency for monitoring the wound, and drug-controlled release by the incorporation of nanoparticles. The incorporation of the nanocarriers aids the drug in permeating into different skin layers, solving the solubility problems of curcumin. The paper by Rancan et al. [10] is related to the production of PVP nanofibrous mats and foils loaded with a poorly soluble antibiotic, ciprofloxacin, for the treatment of topical wound infections. The research has revealed that nanofiber mats reach the highest amount of delivered drug concentration after 6 h, whereas foils maintain a maximum drug concentration over a 24 h period. The treatment has had no effect on the overall skin metabolic activity, but influenced the wound healing process. Importantly, a complete eradication of wound infections with *P. aeruginosa* (10^8^ CFU) could be achieved.

Lian et al. [11] introduced red blood cell membrane-camouflaged ATO-loaded sodium alginate nanoparticles (RBCM-SA-ATO-NPs, RSANs) to relieve the toxicity of ATO while maintaining its efficacy. The average particle size of RSANs has been found to be 163.2 nm with a complete shell-core bilayer structure, and the average encapsulation efficiency is 14.3%. Compared with SANs, RAW 264.7 macrophages reduced the phagocytosis of RSANs by 51%, and the in vitro cumulative release rate of RSANs is 95% at 84 h, which have revealed a prominent sustained release. Furthermore, it has been demonstrated that RSANs have lower cytotoxicity when compared to normal 293 cells and exhibited antitumor effects on both NB4 cells and 7721 cells. In vivo studies have further showed that ATO can cause mild lesions of main organs while RSANs can reduce the toxicity and improve the antitumor effects. Thus, the developed RSANs system has provided a promising alternative for ATO treatment safely and effectively.

Finally, this SI has included the paper by Adeleke et al. [12] that has formulated and evaluated a reconstitutable dry suspension (RDS) containing isoniazid, a first-line antitubercular agent used in the treatment and prevention of TB infection in both children and adults. These formulations have been prepared by direct dispersion emulsification of an aqueous-lipid particulate interphase coupled with lyophilization and dry milling. The dug release behavior has been characterized with an initial burst up to 5 min followed by a cumulative release of 67.88% ± 1.88% (pH 1.2), 60.18% ± 3.33% (pH 6.8), and 49.36% ± 2.83% (pH 7.4) over 2 h. An extended release at pH 7.4 and 100% drug liberation have been achieved within 300 min. RDS has been dispersible and stable in the dried and reconstituted states over 4 months and 11 days respectively, under common storage conditions.

As evident, the SI and the forthcoming e-book demonstrate a range of articles with different research concerns. We hope that both the authors of the papers and ourselves as guest editors have been able to motivate future research in the field of pharmaceutical particulates and membranes for delivering drug and bioactive molecules.

Finally, we would like to acknowledge the contributions made by the authors of each paper irrespective of whether their submissions have been accepted for publication or not, as these have determined the success of this SI and the forthcoming e-book. We also acknowledge the Editorial Office of the Journal *Pharmaceutics* for their continued interest and support in bringing out the SI and the edited e-book.
